# Uncomplicated and Complicated Acute Appendicitis Induce Different Cytokine Patterns

**DOI:** 10.1111/apm.70168

**Published:** 2026-02-19

**Authors:** Tatu Han, Tuomas Borman, Sanja Vanhatalo, Leo Lahti, Eliisa Löyttyniemi, Saija Hurme, Eveliina Munukka, Teemu Kallonen, Antti Hakanen, Maija Hollmén, Paulina Salminen

**Affiliations:** ^1^ Department of Surgery University of Turku Turku Finland; ^2^ Microbe Centre University of Turku and Wellbeing Services County of Southwest Finland Turku Finland; ^3^ Department of Computing University of Turku Turku Finland; ^4^ Research Center for Infections and Immunity Institute of Biomedicine, University of Turku Turku Finland; ^5^ Turku Clinical Microbiome Bank, Department of Clinical Microbiology, Tyks Laboratories Turku University Hospital Turku Finland; ^6^ Department of Biostatistics University of Turku and Turku University Hospital Turku Finland; ^7^ Biocodecs Nordics Espoo Finland; ^8^ InFLAMES Flagship University of Turku and Åbo Akademi University Turku Finland; ^9^ MediCity Research Laboratory University of Turku Turku Finland; ^10^ Department of Digestive Surgery and Urology Turku University Hospital Turku Finland

**Keywords:** appendicitis, IL‐6 prediction, immune response, infection

## Abstract

Although acute appendicitis is one of the most common reasons for emergency surgery, the immunopathogenesis of appendicitis is unclear. The aim of this prospective pre‐defined subgroup analysis study was to characterize serum cytokine profiles and their diagnostic potential in distinguishing between appendicitis severity to offer further insight into the immunopathology of appendicitis. We analyzed 48 cytokines from the serum samples of patients with either uncomplicated or complicated appendicitis. Out of the 113 patients included in the study, 72 patients presented with uncomplicated and 41 patients with complicated acute appendicitis. Compared to uncomplicated appendicitis, complicated appendicitis showed statistically significantly elevated levels in interleukin 6 (IL‐6) (*p* < 0.001), hepatocyte growth factor (HGF) (*p* = 0.003), and monocyte chemoattractant protein 1 (MCP‐1) (*p* < 0.001). IL‐6 had the best performance (area under curve 0.785) in predicting complicated acute appendicitis. The immune response is notably different between complicated and uncomplicated appendicitis, underlining the two different diseases within acute appendicitis. IL‐6 may be utilized as a diagnostic biomarker for complicated appendicitis.

**Trial Registration:** Clinicaltrials.gov NCT03257423

## 1 Introduction

Appendicitis is a major burden to the healthcare system with global incidence of 100–200 cases per 100,000 person‐years [1] and one of the most common reasons for emergency surgery across the globe [2]. Based on epidemiological trends [3], microbiological studies [4, 5], histopathology [6], clinical and registry studies [7, 8, 9] we now know that uncomplicated and complicated acute appendicitis are actually two different diseases having a major impact on the treatment alternatives. To date, the pathogenesis of these two different forms of acute appendicitis is still unclear [10]. Local inflammation or appendiceal obstruction causing bacterial overgrowth in the appendiceal lumen has been one of the hypotheses for the pathogenesis of appendicitis, but accumulating evidence suggests primary bacterial infection as the initiating event of the disease [11, 12]. Acute appendicitis and its severity are associated with an abundance of bacteria from the phylum Fusobacteria [11, 12, 13] and the most common bacteria found in intraoperative cultures is reported to be *Escherichia coli* and *Bacteroides fragilis* [13, 14]. Instead of the increase of a single bacterial species, a shift in the appendiceal microbiome is hypothesized to lead to the infection [11, 15]. A recent systematic review evidenced that IgE‐mediated allergy may have a role in the development of uncomplicated acute appendicitis [16], thus questioning the hypothesis of microbiological origin of acute appendicitis.

The most common biomarkers used in diagnosing acute appendicitis are C‐reactive protein (CRP) and white blood cell count (WBC) [17, 18]. For the past few decades there has been active research in identifying more accurate and better biomarkers, the most promising being interleukin 6 (IL‐6) [19, 20], a cytokine that is secreted by macrophages in response to specific microbial molecules. A recent study with pediatric patients shows good performance of IL‐6 in differentiating between uncomplicated and complicated acute appendicitis [21]. Other studies focusing on appendicitis immunopathology show that polymorphism of the IL‐6 gene is associated with appendicitis severity [22] and several studies report different cytokine profiles between uncomplicated and complicated appendicitis [17, 23, 24] implicating that appendicitis severity may be associated with different immunological responses [25].

Based on multiple randomized controlled trials (RCTs), antibiotics alone have been shown to be a feasible and safe alternative for the treatment of computed tomography (CT) diagnosed uncomplicated acute appendicitis [8, 25, 26, 27] and even symptomatic therapy may be sufficient [28, 29]. To optimize both the diagnostics and treatment of acute appendicitis, we need to bridge the existing knowledge gaps of understanding the different pathophysiology of both uncomplicated and complicated appendicitis. Understanding immune responses within the whole spectrum of acute appendicitis could offer insights to improved diagnostics and management of appendicitis. The aim of this study was to identify serum cytokine profiles of uncomplicated and complicated acute appendicitis in a prospective observational cohort study assessing the possible association of different cytokines with appendicitis severity. This study showed that the systemic immune response is notably different between complicated and uncomplicated appendicitis, offering further insight into the immunopathology of acute appendicitis and underlining the two different diseases within acute appendicitis.

## 2 Methods

### 2.1 Study Design

This study was a prospective pre‐defined subgroup analysis of cytokine data as a part of the prospective MAPPAC (Microbiology APPendicitis Acuta) observational cohort study conducted in concurrence with the randomized controlled trials APPAC II (APPendicitis Acuta, ClinicalTrials.gov NCT03236961) and APPAC III (NCT03234296) using the same patient enrollment population collected at the Turku University Hospital, Turku, Finland. This study was a predefined single‐center arm of the MAPPAC study at Turku University Hospital aiming to evaluate microbiological and immunological aspects associated with appendicitis severity [30]. Briefly, APPAC II was a multi‐center RCT comparing oral antibiotic monotherapy with intravenous antibiotic therapy followed by oral antibiotics in CT‐confirmed acute uncomplicated appendicitis [31]. APPAC III was a multi‐center, double‐blind, RCT comparing antibiotic therapy with placebo in CT‐confirmed acute uncomplicated appendicitis [32]. All patients included in these studies gave written informed consent. The ethics committee of the Hospital District of Southwest Finland approved trial protocols (Turku University Hospital, approval number ATMK:142/1800/2016) and the Finnish Medicines Agency (Fimea), and the trial was registered in ClinicalTrials.gov (NCT03257423). The trial was performed in accordance with relevant guidelines and regulations such as the Declaration of Helsinki.

### 2.2 The Aim

The aim of this study was to assess whether acute appendicitis influences the systemic immunological response and if there are any differences in immunological responses between uncomplicated and complicated acute appendicitis. Based on the two different diseases within acute appendicitis, we hypothesized that systemic immunological responses would differ between appendicitis severities.

### 2.3 Study Participants

Patients admitted to the emergency department with CT and clinically or histopathologically confirmed diagnosis of uncomplicated or complicated acute appendicitis with available serum samples between April 11th, 2017, and September 2nd, 2019, were included in this study. Patients presenting with a suspected recurrent appendicitis were not eligible for study enrollment. We defined recurrent appendicitis as a new episode of appendicitis occurring after initial successful non‐operative therapy for uncomplicated acute appendicitis. In patients undergoing non‐operative treatment, the diagnosis of appendicitis severity was confirmed by CT and no hospital re‐admission within 30‐days. In patients undergoing appendectomy (patients with complicated acute appendicitis or not eligible for non‐operative treatment), the diagnosis was determined by CT, surgery, and histopathology. The CT criteria for uncomplicated acute appendicitis included appendiceal diameter exceeding 6 mm with at least one of the following findings: abnormal contrast enhancement of the appendiceal wall, inflammatory edema, or fluid collections around the appendix. Operative or histopathological findings of appendicolith, gangrene, perforation, abscess, or tumor were classified as complicated acute appendicitis. To validate the accuracy of the differential diagnosis between uncomplicated and complicated acute appendicitis, all patients were assessed by two investigators (Suvi Sippola and Jussi Haijanen) unaware of the other's evaluation. In cases of disagreement, the clinical diagnosis was reviewed by a third investigator (P.S.) [25].

### 2.4 Sample Collection and Preparation

From patients evaluated for enrollment in APPAC II or III RCTs, serum samples were collected at the emergency department before any treatment and stored at −70°C. Concentrations of cytokines were measured from serum samples using Bio‐Plex Pro Human 48‐Plex (Bio‐Rad Laboratories Inc.; California; USA) according to the manufacturer's protocol. The cytokines, chemokines, and growth factors included in the panel are described in detail in Table 1.

**TABLE 1:** Analyzed cytokines and their abbreviations.

	Cytokine						
1	Basic fibroblast growth factor (bFGF)	13	Interleukin 1 beta (IL‐1β)	25	Interleukin 12 p40 (IL‐12p40)	37	Monocyte chemoattractant protein 1 (MCP‐1)
2	Beta nerve growth factor (β‐NGF)	14	Interleukin 1 receptor antagonist (IL‐1ra)	26	Interleukin 12 p70 (IL‐12p70)	38	Monocyte chemotactic protein 3 (MCP‐3)
3	Cutaneous T cell‐attracting chemokine (CTACK)	15	Interleukin 2 (IL‐2)	27	Interleukin 13 (IL‐13)	39	Monokine induced by gamma interferon (MIG)
4	Eotaxin (CCL‐11)	16	Interleukin 2 receptor alpha (IL‐2Rα)	28	Interleukin 15 (IL‐15)	40	Platelet derived growth factor BB (PDGF‐BB)
5	Granulocyte colony‐stimulating factor (GCSF)	17	Interleukin 3 (IL‐3)	29	Interleukin 16 (IL‐16)	41	Regulated on activation, normal T cell expressed and secreted (RANTES)
6	Granulocyte‐macrophage colony‐stimulating factor (GM‐CSF)	18	Interleukin 4 (IL‐4)	30	Interleukin 17A (IL‐17A)	42	Stem cell factor (SCF)
7	Growth‐regulated oncogene alpha (GRO‐α)	19	Interleukin 5 (IL‐5)	31	Interleukin 18 (IL‐18)	43	Stem cell growth factor beta (SCGF‐β)
8	Hepatocyte growth factor (HGF)	20	Interleukin 6 (IL‐6)	32	Leukemia inhibitory factor (LIF)	44	Stromal cell‐derived factor 1 alpha (SDF‐1α)
9	Interferon alpha 2 (IFN‐α2)	21	Interleukin 7 (IL‐7)	33	Macrophage colony‐stimulating factor (MCSF)	45	TNF‐related apoptosis‐inducing ligand (TRAIL)
10	Interferon gamma (IFN‐γ)	22	Interleukin 8 (IL‐8)	34	Macrophage inflammatory protein 1 alpha (MIP‐1α)	46	Tumor necrosis factor alpha (TNF‐α)
11	Interferon gamma‐induced protein 10 (IP‐10)	23	Interleukin 9 (IL‐9)	35	Macrophage inflammatory protein 1 beta (MIP‐1β)	47	Tumor necrosis factor beta (TNF‐β)
12	Interleukin 1 alpha (IL‐1α)	24	Interleukin 10 (IL‐10)	36	Macrophage migration inhibitory factor (MIF)	48	Vascular endothelial growth factor (VEGF)

### 2.5 Statistical Analysis

All statistical analyses were conducted using R 4.3.1 [33]. Beta diversity analyses were performed with the R/Bioconductor package mia (version 1.9.20) [34]; *p*‐values were adjusted using the Benjamini‐Hochberg FDR (false discovery rate) correction [35], and adjusted *p*‐values less than 0.05 were considered statistically significant.

Values that exceeded the cytokine‐specific thresholds were truncated to 0.5*lower and 1.5*upper threshold respectively [36, 37]. To reduce the batch effect caused by cytokine plates, data was log10‐transformed and then normalized cytokine plate‐wise with Z‐transformation before statistical analysis. The ability to reduce the plate effect with normalization was evaluated by modeling the associations between total cytokine concentrations, diagnosis and cytokine plates using linear mixed effects models. In the two models built with lme4 R package (version 1.1–34) [38], the response variable was the total cytokine level, and diagnosis was modeled as a random effect. The second model included the cytokine plate as a fixed effect. The models were compared with Chi‐squared test to assess the effect of cytokine plate. The association between total cytokine concentrations and diagnosis was quantified with the Wilcoxon rank sum test. Associations between cytokine profile and covariates were visualized using redundancy analysis (RDA) by plotting weighted average site scores. The covariates included age, body mass index (BMI), CRP, cytokine plate, diagnosis, sex, body temperature, leukocytes, and neutrophils. Associations between the covariates and cytokine profile were analyzed by using permutational multivariate analysis of variance (PERMANOVA) with 999 random permutations and the homogeneity of groups were examined by using PERMDISP2 procedure along with analysis of variance (ANOVA). The Wilcoxon rank sum test was applied to test if concentrations of individual cytokines differed between the appendicitis forms, and to estimate medians.

Data was split randomly into training and test sets (70:30 ratio). An extreme gradient boosting (XGBoost) model with leave‐one‐out cross‐validation (LOOCV) was fitted by using the caret R package (version 6.0–94) [39]. The hyperparameters were tuned with Bayesian optimization by utilizing the R package mlrMBO (version 1.1.5.1), optimizing for ROC‐AUC [40]. The tuned model (hyperparameters: nrounds = 979, max_depth = 9, eta = 0.2038419, gamma = 6.856464, colsample_bytree = 0.6485451, min_child_weight = 3.195767, subsample = 0.5937414) was used to predict appendicitis severity in the test set. We applied the same datasets to fit logistic regression models. For each cytokine, a model was trained using the caret R package (version 6.0–94) [39]. Models were evaluated using leave‐one‐out cross‐validation. The model with the highest AUC‐ROC (area under the receiver operating characteristic curve) in the training set was used to predict appendicitis severity in the test set.

## 3 Results

**FIGURE 1:**
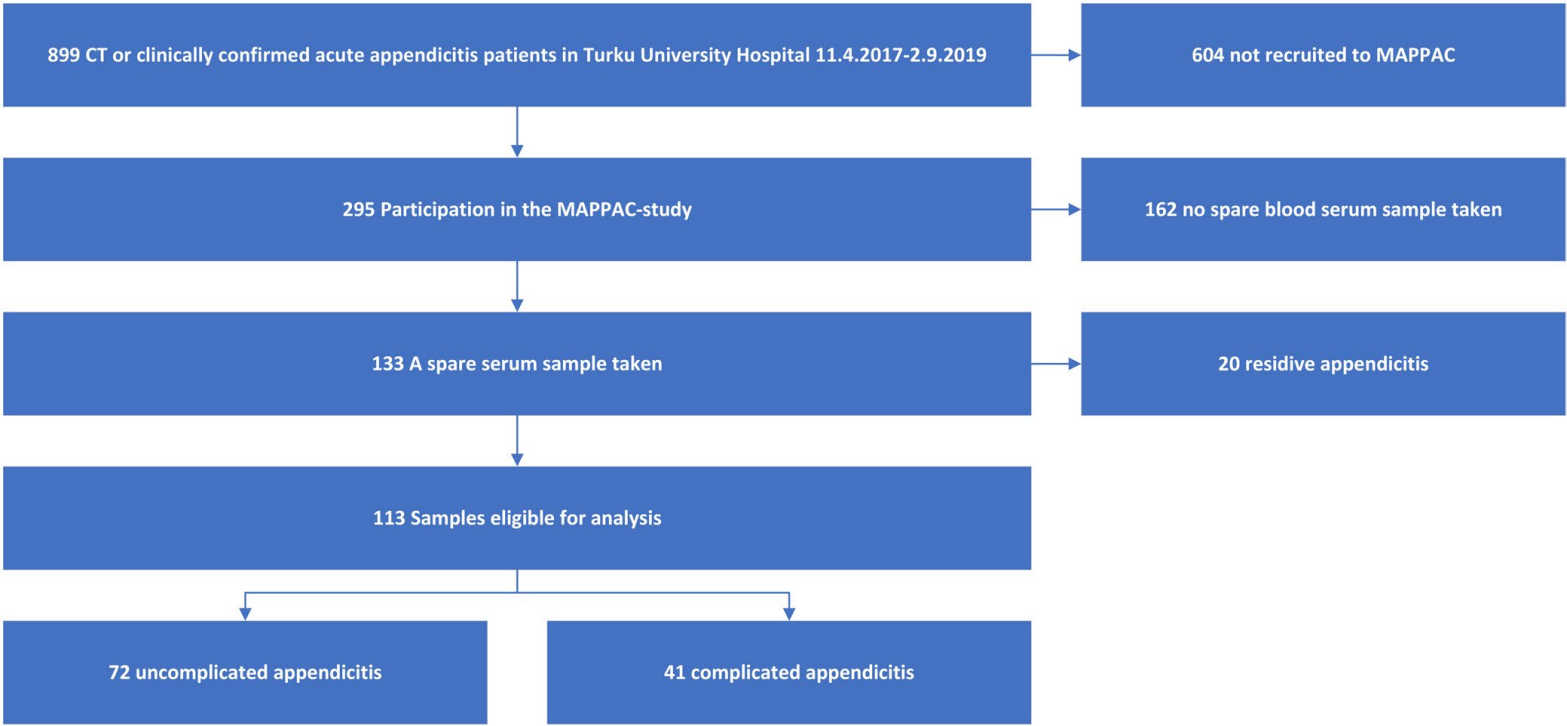
Study flowchart.

Between April 11th, 2017, to September 2nd, 2019, there were a total of 889 patients with CT and clinically or histopathologically confirmed diagnosis of uncomplicated or complicated acute appendicitis diagnosed at the emergency department of Turku University Hospital. Out of these patients, 295 (33%) participated in the MAPPAC‐study, and 133 patients had an additional serum sample taken. After the exclusion of 20 patients with recurrent appendicitis, a total of 113 patients were eligible for this study. Out of these 113 patients, 47 (41.6%) were female, 66 (58.4%) male, median age was 36 years (min 18, max 75), 72 (64%) had uncomplicated acute appendicitis and 41 (36%) had complicated acute appendicitis. Figure 1 shows the patient flow and Table 2 baseline characteristics.

**TABLE 2:** Patient characteristics: Complicated versus uncomplicated acute appendicitis.

	Uncomplicated appendicitis^a^	Complicated appendicitis^b^
*N*	72	41
Age, years, (median [MAD])	33.5 [11.9]	42.0 [18.4]
BMI, kg/m^2^, (median [MAD])	27.4 [4.6]	26.6 [5.2]
CRP, mg/L, (median [MAD])	25.5 [22.3]	35.0 [44.5]
WBC, 10^9^/L, (median [MAD])	11.3 [4.1]	14.2 [2.7]
Neut, 10^9^/L, (mean [SD])	8.6 [3.1]	12.7 [2.6]
Sex‐female (*N*)	29	18
Sex‐male (*N*)	43	23
Temp, °C, (mean [SD])	37.5 [0.6]	37.3 [0.6]

Abbreviations: BMI, body mass index; CRP, C‐reactive protein; MAD, median absolute value; Neut, neutrophile count; SD, standard deviation; Temp, body temperature; WBC, white blood cell count.

^a^ Uncomplicated appendicitis: CT criteria for uncomplicated acute appendicitis included appendiceal diameter exceeding 6 mm with at least one of the following findings: abnormal contrast enhancement of the appendiceal wall, inflammatory edema, or fluid collections around the appendix.

^b^ Complicated appendicitis: Operative or histopathological findings of appendicolith, gangrene, perforation, abscess, or tumor were classified as complicated acute appendicitis.

### 3.1 Cytokine Levels Associated With Appendicitis Severity

**FIGURE 2:**
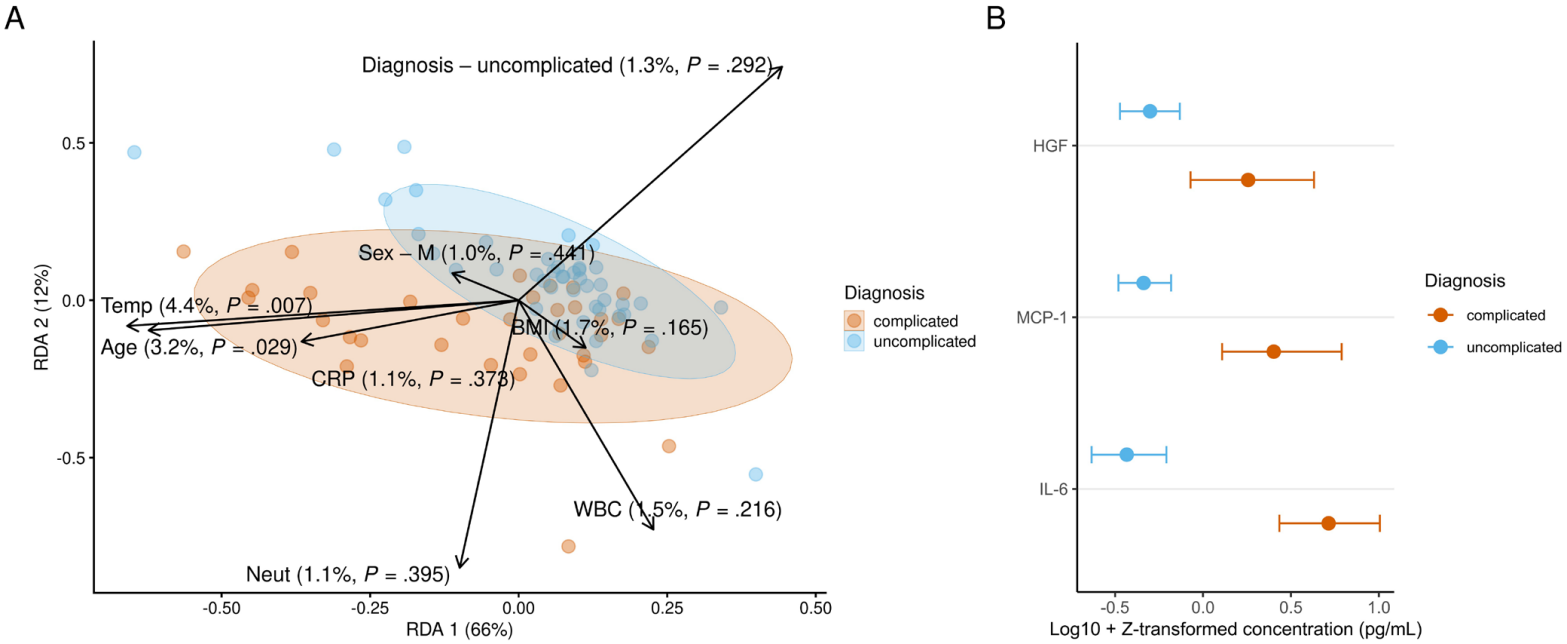
Redundancy analysis and concentrations of selected cytokines. (A) Redundancy analysis (RDA) of cytokine profiles with covariates: Age, body mass index (BMI), C‐reactive protein (CRP), body temperature (temp), white blood cell (WBC) and neutrophil (Neut) counts, sex, and diagnosis. Percentages and *p*‐values on arrows denote the explained variance and significance per covariate. Percentages on axis denote the explained variance along the corresponding principal coordinate. (B) Estimated medians of statistically significant cytokines by diagnosis. The estimation was performed with the Wilcoxon test. (values log10‐ and Z‐transformed per cytokine plate‐wise).

In the 113 serum samples, plate‐specific systematic differences were not observed after normalization, which was hence ignored in the subsequent analyses. The total cytokine concentration did not differ between uncomplicated and complicated acute appendicitis. In the redundancy analysis, there were significant associations between the cytokine profiles and covariates (explained variance 19.4%; PERMANOVA; adjusted *p* = 0.003). Of the individual covariates, body temperature and age were the only two statistically significant factors (Figure 2a).

Principal component analysis (PCA) yielded results consistent with those of the RDA (Figure S1). There were significant differences in three individual cytokines of Interleukin 6 (IL‐6), Monocyte chemoattractant protein 1 (MCP‐1), and Hepatocyte growth factor (HGF) (adjusted *p* < 0.05) and all 3 cytokines showed higher levels in the serum of patients diagnosed with complicated acute appendicitis (Figure 2b, Table 3).

**TABLE 3:** The three cytokines with statistically significant differences between complicated and uncomplicated acute appendicitis.

	*p*‐value (adjusted)^a^	Median level in uncomplicated appendicitis	Median level in complicated appendicitis	Effect size	Difference of medians	Confidence interval (95%)
IL‐6	< 0.001	−0.567	0.716	0.108	1.283	0.809–1.530
MCP‐1	0.001	−0.413	0.434	−0.072	0.847	0.356–1.072
HGF	0.042	−0.311	0.231	−0.266	0.542	0.189–0.842

^a^ Significance was tested with the Wilcoxon test.

**FIGURE 3:**
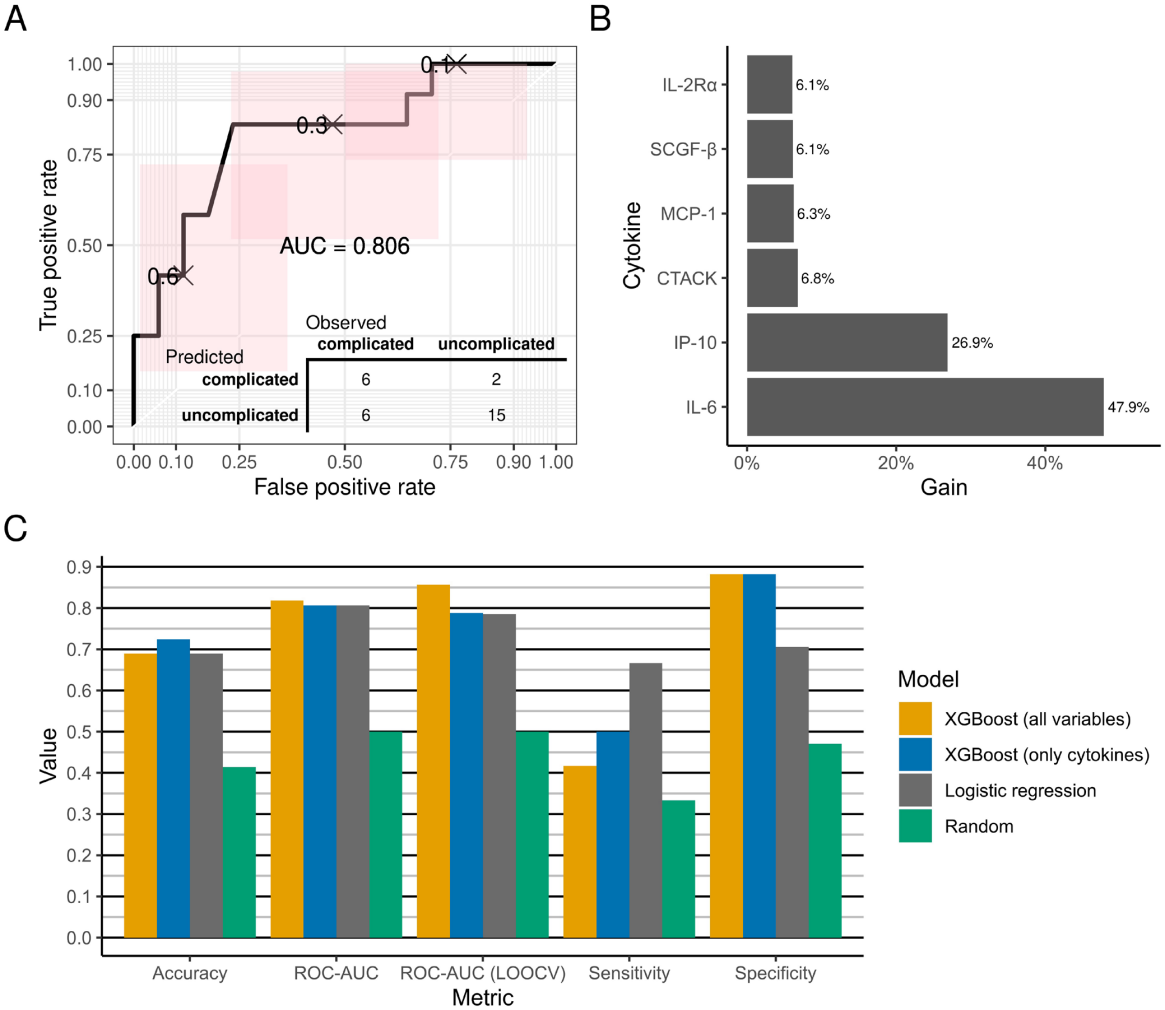
Analyzing the predictability of appendicitis severity using cytokines. (A) A ROC curve depicting the prediction of appendicitis severity using the test set samples. The bottom corner includes a confusion matrix, illustrating how the predictions from the test set were distributed across the observed outcomes. (B) XGBoost feature importances indicating the impact of each cytokine on prediction performance. (C) Machine learning models were evaluated, comparing accuracy, ROC‐AUC (for both test and training sets), sensitivity, and specificity. This included XGBoost models with only cytokines, an extended version with all covariates (appendix), a logistic regression model, and a baseline of random guessing. Identical datasets were used for training and testing.

Due to the observed differences in cytokine levels between complicated and uncomplicated appendicitis, we evaluated whether clinical diagnosis could be predicted based on cytokine levels. In XGBoost model receiver operating characteristics ROC‐AUC for the test set was 0.806 (ROC‐AUC for train set 0.788) with sensitivity and specificity of 0.500 and 0.882, respectively (Figure 3).

**FIGURE 4:**
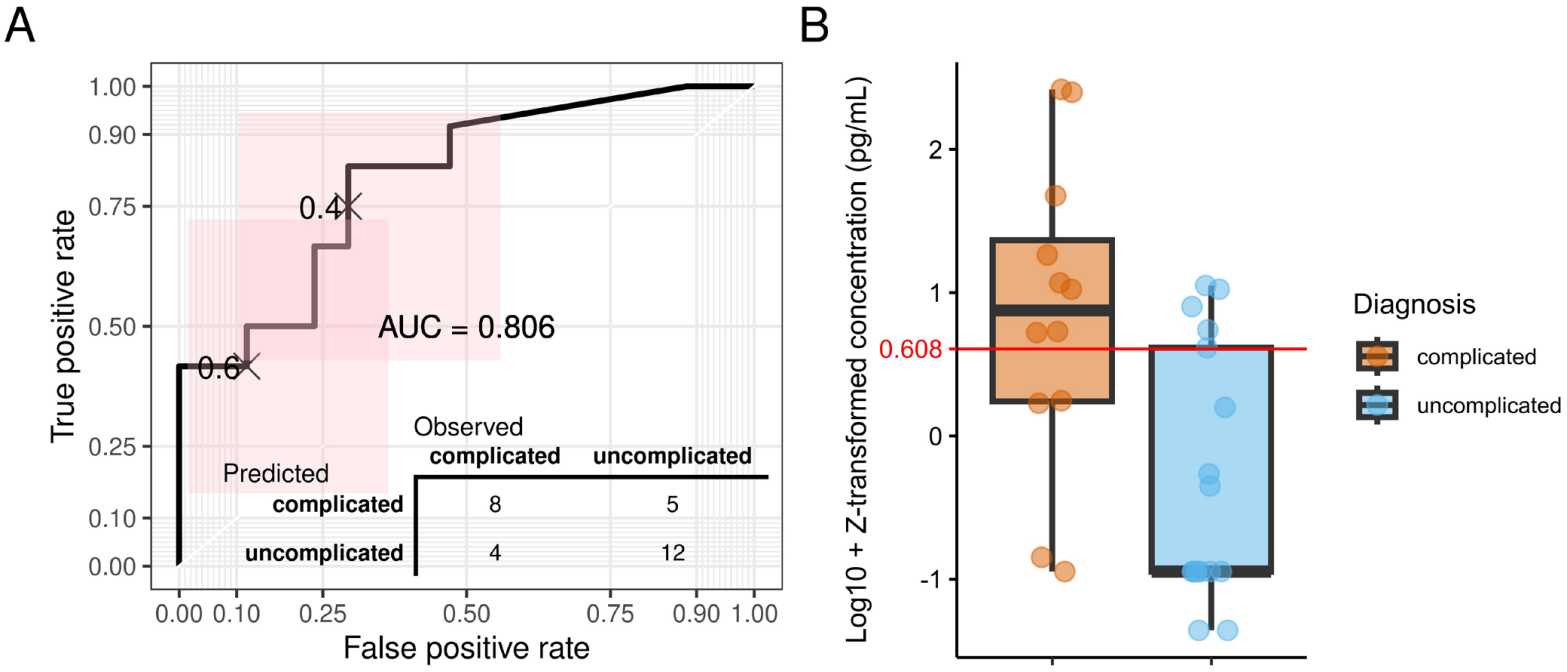
The logistic regression model fitted using IL‐6 as a biomarker. (A) A ROC curve depicting the predictions made on the test set samples. The bottom right corner includes a confusion matrix, illustrating how the predictions from the test set were distributed across the observed outcomes. (B) The distributions of IL‐6 concentrations for different appendicitis forms along with the model's decision threshold (0.608).

In the logistic regression model, IL‐6 showed the best performance in the training data, and it was thus used for predicting outcomes in the test set. The logistic regression model demonstrated ROC‐AUC of 0.806 (ROC‐AUC for train set 0.785), achieving a sensitivity of 0.667 and a specificity of 0.706 (Figure 4). The inclusion of additional covariates (sex, temperature, age, CRP, BMI, Neut, and WBC) in XGBoost demonstrated that the ROC‐AUC for the test set was 0.882 (ROC‐AUC for train set 0.857), sensitivity and specificity being 0.417 and 0.826, respectively (Figure S2).

## 4 Discussion

In this prospective observational pre‐defined study, complicated acute appendicitis was statistically significantly associated with increased levels of three serum cytokines (IL‐6, HGF, and MCP‐1) compared to uncomplicated acute appendicitis. This difference of the systemic immune response between complicated and uncomplicated appendicitis further underlines and supports the distinction between these two different forms of appendicitis severity. Out of these three cytokines, IL‐6 had the best performance in predicting complicated acute appendicitis.

Appendicitis is associated with a systemic inflammatory response usually causing fever and elevating serum WBC and CRP levels [41, 42, 43, 44]. One study reported elevated pro‐inflammatory cytokines in perforated compared to non‐perforated appendicitis [45], while another study observed an anti‐inflammatory cytokine profile in acute appendicitis [46] in concurrence with this study. While we assumed that the cytokine profile in complicated appendicitis would be primarily pro‐inflammatory, our analysis suggests that the complicated form of appendicitis can increase both pro‐ and anti‐inflammatory responses. Out of the three cytokines with statistically significantly higher serum concentrations in complicated appendicitis, there was one pro‐inflammatory cytokine (IL‐6) and two mainly anti‐inflammatory cytokines (HGF, MCP‐1). This suggests that while complicated appendicitis is clinically a more severe condition compared to uncomplicated appendicitis, it also induces a stronger inflammatory response observable in the subgroup analysis.

Our research findings suggest that machine learning may show potential in predicting the type of appendicitis based on cytokine levels. It is important to highlight that the inclusion of additional covariates (sex, temperature, age, CRP, BMI, Neutrophil count, and WBC) did not notably enhance the model suggesting that the primary diagnostic signal resides within the cytokines. CT is considered the golden standard of diagnosing acute appendicitis and both low‐dose and standard CT protocols have similar accuracy of differentiating between uncomplicated and complicated appendicitis [47, 48]. Avanesov et al. combined radiological and clinical factors including age, body temperature, duration of symptoms, appendix diameter, presence of periappendiceal fluid, extraluminal air and a perityphilis abscess to form APSI‐index in the purpose of better management of acute appendicitis in adults. For predicting complicated appendicitis this index has a positive predictive value of 92% and negative predictive value of 83% [49]. Another similar scoring model referred as Scoring System of Appendicitis Severity (SAS) had a sensitivity of 83.6% and a negative predictive value of 85.0%. Scheijmans et al. developed an improved version of SAS called SAS 2.0 [50] with high accuracy for predicting complicated appendicitis adding to the clinical tools in improving appendicitis severity diagnostics [51].

In addition to our complex machine learning model, we also fitted a simpler logistic regression model for its ease of interpretation. Our analyses indicated that IL‐6 may be a feasible biomarker for predicting the form of appendicitis. The model exhibited a ROC‐AUC value comparable to that observed in the complex model, suggesting that IL‐6 concentration includes a significant portion of the information regarding patient diagnosis status. While IL‐6 has good performance, it does not reach the level of CT when predicting the form of appendicitis. This finding is in line with previous studies made in the pediatric population. Ozguner et al. reported a cut‐off point of 8 pg/mL with 76.5% sensitivity and 71.4% specificity when identifying appendicitis from nonspecific abdominal pain [19]. Another study in 2020 proposed a cut‐off value of 26.43 pg/mL with 77.4% sensitivity and 58.1% specificity separating complicated appendicitis from the uncomplicated form [20]. Arredondo Montero et al. also reported that IL‐6 shows good performance in discerning between complicated and uncomplicated pediatric acute appendicitis [21]. It is worth noting that IL‐6 emerged as the most important cytokine in our complex machine learning model. As a result of the good performance of IL‐6 in predicting the form of appendicitis, combining this new biomarker to other clinical and radiological data may yield a more accurate instrument of discriminating the different forms of appendicitis and thus could decrease unnecessary appendectomies in uncomplicated cases.

There are several limitations in this study. First, the serum samples were available in only 113 (13%) patients out of the evaluated cohort of 899 patients. However, the prospective study population with available serum samples was relatively large compared to previous studies. In addition, the number of patients with complicated acute appendicitis was small and the majority of these were patients presenting with an appendicolith preventing any subgroup analysis of the different forms of complicated acute appendicitis (gangrene, perforation, and periappendicular abscess). A second major limitation is the lack of appendiceal luminal microbiological data. The aim was to include microbiological data in the analyses, but there were only 29 patients with available intraluminal microbiota swab with uneven distribution between appendicitis severity preventing any comparison to cytokine profiles. Thirdly, we were not able to define IL‐6 cut‐off points since the concentrations were relative to cytokine plates. One strength of this study was the comprehensive number of cytokines analyzed, which enabled us to observe the differences between these two forms of the disease more broadly identifying cytokine pattern differences. In addition, the prospective patient cohort and as accurate as possible clinical differential diagnosis between complicated and uncomplicated acute appendicitis are major strengths of the study.

## 5 Conclusions

The systemic immune response is notably different between complicated and uncomplicated appendicitis, offering further insight into the immunopathology of acute appendicitis and underlining the two different diseases within acute appendicitis. IL‐6 was the best performing cytokine differentiating between complicated and uncomplicated acute appendicitis.

## Funding

TH was funded by Turku University Foundation and Government hospital research grant. TB was funded by UTUGS graduate school. LL was funded by Academy of Finland 330887. PS received funding from Academy of Finland and Sigrid Jusélius Foundation. The funders had no role in study design, data collection and analysis, decision to publish, or preparation of the manuscript.

## Ethics Statement

This study was performed in line with the principles of the Declaration of Helsinki. Approval was granted by the Ethics Committee of the Hospital District of Southwest Finland (Turku University Hospital, approval number ATMK:142/1800/2016).

## Consent

Informed consent was obtained from all individual participants included in the study.

## Conflicts of Interest

The authors of this manuscript have the following: competing interests: Paulina Salminen: Lecture fees: Novo Nordisk, Ethicon, BD. Research grants: Sigrid Jusélius Foundation, Academy of Finland, European Research Council. Data safety monitoring committee: GT Metabolic Solutions.

## Data and Code availability

The datasets used and/or analysed during the current study are available from the corresponding author on reasonable request. The code used in this study can be found in Zenodo: https://zenodo.org/records/10220197.

## Supporting information


**Figure S1:** Principal component analysis (PCA). PCA was conducted to log10 and Z‐transformed values with the R package scater (version 1.29.4) (52).

**Figure S2:** Data including cytokines and covariates (age, body mass index (BMI), C‐reactive protein (CRP), sex, body temperature, white blood cell (WBC), and neutrophile (Neut) count) was split randomly into training and test sets (70:30 ratio) (52). The cytokine data was log10 and Z‐transformed cytokine plate‐wise. Numeric covariates were log10 and Z‐transformed, and categorical covariates were one‐hot‐encoded. Extreme gradient boosting (XGBoost) model was fitted with leave‐one‐out cross‐validation by using the caret R package (39). The hyperparameters were tuned with Bayesian optimization by utilizing R package mlrMBO, optimizing for ROC‐AUC. The tuned model (hyperparameters: nrounds = 1352, max_depth = 6, eta = 0.1588734, gamma = 4.381891, colsample_bytree = 0.3683497, min_child_weight = 1.846714, subsample = 0.2152322) was used to predict appendicitis forms in the test set. ROC‐AUC for the test set was 0.819 (ROC AUC for train set 0.857) sensitivity and specificity being 0.417 and 0.882, respectively. (A) A ROC curve depicting the predictions made on the test set samples. The bottom corner includes a confusion matrix, illustrating how the predictions from the test set were distributed across the observed outcomes. (B) XGBoost feature importances indicating the impact of each cytokine and covariate on prediction performance.

## Data Availability

The data that support the findings of this study are available from the corresponding author upon reasonable request.
