# Supplementary material for: Uncomplicated and Complicated Acute Appendicitis Induce Different Cytokine Patterns
Source: APMIS. 2026 Feb 19;134(2):e70168. doi: 10.1111/apm.70168 (PMC12917868; doi:10.1111/apm.70168)
Supplement: Supplementary file 1 — Figure S1: Principal component analysis (PCA). PCA was conducted to log10 and Z‐transformed values with the R package scater (version 1.29.4) (52). [file APM-134-0-s001.pdf]

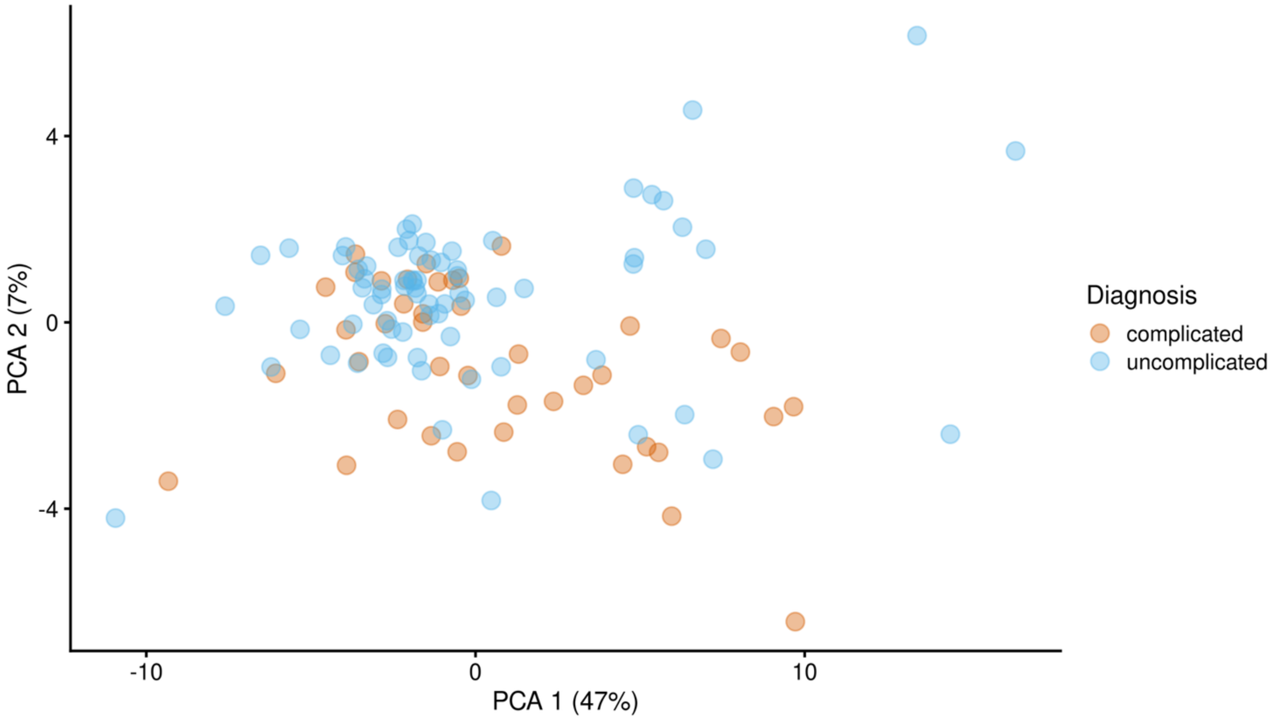

Supplementary Figure 1. Principal component analysis (PCA). PCA was conducted to log10 and Z-transformed values with the R package scater (version 1.29.4) (52).
