# Supplementary material for: Uncomplicated and Complicated Acute Appendicitis Induce Different Cytokine Patterns
Source: APMIS. 2026 Feb 19;134(2):e70168. doi: 10.1111/apm.70168 (PMC12917868; doi:10.1111/apm.70168)
Supplement: Supplementary file 2 — Figure S2: Data including cytokines and covariates (age, body mass index (BMI), C‐reactive protein (CRP), sex, body temperature, white blood cell (WBC), and neutrophile (Neut) count) was split randomly into training and test sets (70:30 ratio) (52). The cytokine data was log10 and Z‐transformed cytokine plate‐wise. Numeric covariates were log10 and Z‐transformed, and categorical covariates were one‐hot‐encoded. Extreme gradient boosting (XGBoost) model was fitted with leave‐one‐out cross‐validation by using the caret R package (39). The hyperparameters were tuned with Bayesian optimization by utilizing R package mlrMBO, optimizing for ROC‐AUC. The tuned model (hyperparameters: nrounds = 1352, max_depth = 6, eta = 0.1588734, gamma = 4.381891, colsample_bytree = 0.3683497, min_child_weight = 1.846714, subsample = 0.2152322) was used to predict appendicitis forms in the test set. ROC‐AUC for the test set was 0.819 (ROC AUC for train set 0.857) sensitivity and specificity being 0.417 and 0.882, respectively. (A) A ROC curve depicting the predictions made on the test set samples. The bottom corner includes a confusion matrix, illustrating how the predictions from the test set were distributed across the observed outcomes. (B) XGBoost feature importances indicating the impact of each cytokine and covariate on prediction performance. [file APM-134-0-s002.pdf]

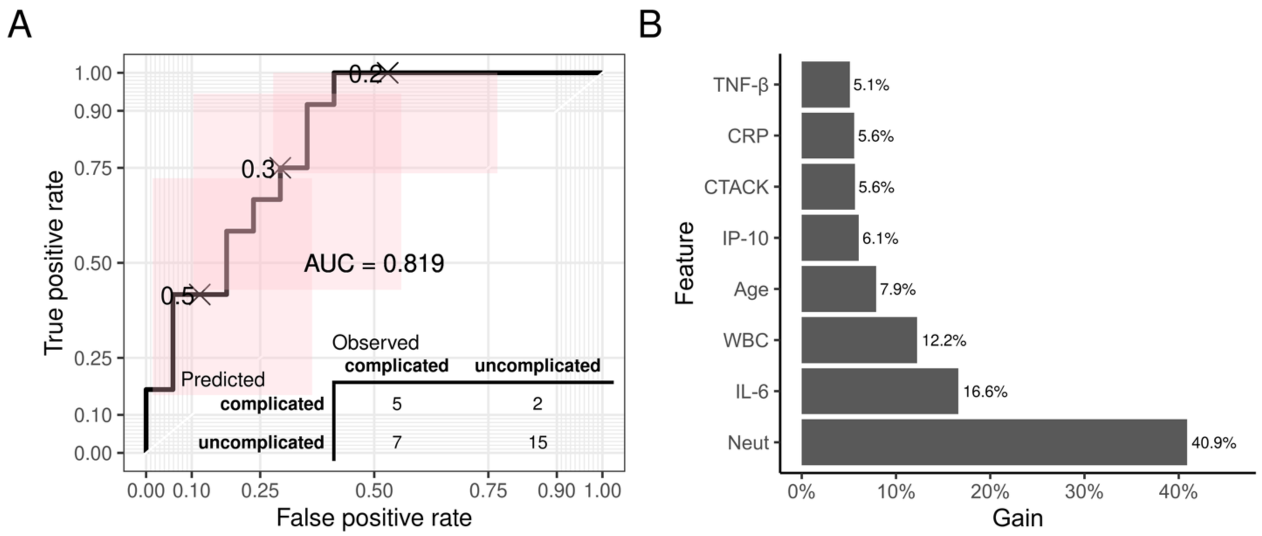

Supplementary Figure 2. Data including cytokines and covariates (age, body mass index (BMI), C-reactive protein (CRP), sex, body temperature, white blood cell (WBC), and neutrophile (Neut) count) was split randomly into training and test sets (70:30 ratio) (52). The cytokine data was log10 and Z-transformed cytokine plate-wise. Numeric covariates were log10 and Z-transformed, and categorical covariates were one-hot-encoded. Extreme gradient boosting (XGBoost) model was fitted with leave-one-out cross-validation by using the caret R package (39). The hyperparameters were tuned with Bayesian optimization by utilizing R package mlrMBO, optimizing for ROC-AUC. The tuned model (hyperparameters: nrounds=1352, max_depth=6, eta=0.1588734, gamma=4.381891, colsample_bytree=0.3683497, min_child_weight=1.846714, subsample=0.2152322) was used to predict appendicitis forms in the test set. ROC-AUC for the test set was 0.819 (ROC-AUC for train set 0.857) sensitivity and specificity being 0.417 and 0.882, respectively. A. A ROC curve depicting the predictions made on the test set samples. The bottom corner includes a confusion matrix, illustrating how the predictions from the test set were distributed across the observed outcomes. B. XGBoost feature importances indicating the impact of each cytokine and covariate on prediction performance.
